# Plant-Based Diet as a Strategy for Weight Control

**DOI:** 10.3390/foods10123052

**Published:** 2021-12-08

**Authors:** Stanislava Ivanova, Cédric Delattre, Diana Karcheva-Bahchevanska, Niko Benbasat, Vanya Nalbantova, Kalin Ivanov

**Affiliations:** 1 Department of Pharmacognosy and Pharmaceutical Chemistry, Faculty of Pharmacy, Medical University-Plovdiv, 4002 Plovdiv, Bulgaria; diana.karcheva@mu-plovdiv.bg (D.K.-B.); niko.benbasat@mu-plovdiv.bg (N.B.); vanya.nalbantova@mu-plovdiv.bg (V.N.); kalin.ivanov@mu-plovdiv.bg (K.I.); 2 Institut Pascal, Université Clermont Auvergne, Clermont Auvergne INP, CNRS, 63000 Clermont-Ferrand, France; cedric.delattre@uca.fr; 3 Institut Universitaire de France (IUF), 1 Rue Descartes, 75005 Paris, France; 4 Department of Pharmacognosy, Faculty of Pharmacy, Medical University of Sofia, 1000 Sofia, Bulgaria

**Keywords:** obesity, globesity, vegan, plant-based diet, vegetarian, weight control, weight loss, obesity management

## Abstract

According to the World Health Organization, obesity has nearly tripled since the 1970s. Obesity and overweight are major risk factors for cardiovascular diseases, diabetes, inflammatory-mediated diseases, and other serious medical conditions. Moreover, recent data suggest that obesity, overweight, diabetes, and cardiovascular diseases are risk factors for COVID-19-related mortality. Different strategies for weight control have been introduced over the last two decades. Unfortunately, these strategies have shown little effect. At the same time, many studies show that plants might be the key to a successful strategy for weight control. Following the PRISMA guidelines for conducting systematic reviews, a search was conducted in PubMed, Web of Science, Scopus, and Embase using the following keywords: obesity, globesity, vegan, plant-based diet, etc. Our results show that vegan diets are associated with improved gut microbiota symbiosis, increased insulin sensitivity, activation of peroxisome proliferator-activated receptors, and over-expression of mitochondrial uncoupling proteins. The key features of this diet are reduced calorie density and reduced cholesterol intake. The combination of these two factors is the essence of the efficiency of this approach to weight control. Our data suggest that plant-based/vegan diets might play a significant role in future strategies for reducing body weight.

## 1. Introduction

According to the World Health Organization (WHO), obesity has nearly tripled since the 1970s [1]. Nowadays, it is spread almost worldwide, reaching pandemic levels [1,2,3,4,5,6,7,8,9,10]. More than 1.9 billion adults are overweight, 650 million of whom are obese. Moreover, children are also affected. The WHO reports that 39 million children under the age of 5 are overweight or obese in 2020 [1].

Although obesity is considered to be preventable, to date, there is no reduction in the prevalence of individuals with overweight/obesity [7].

Obesity and overweight have huge impacts on health [2,3,4,5,6,7,8,9,10]. The conditions are defined as “abnormal/excessive fat accumulation that may impair health”. Overweight adults are individuals with a Body Mass Index (BMI) greater than or equal to 25 kg/m^2^. Adults with a BMI equal to/greater than 30 kg/m^2^ are considered obese.

**Figure 1:**
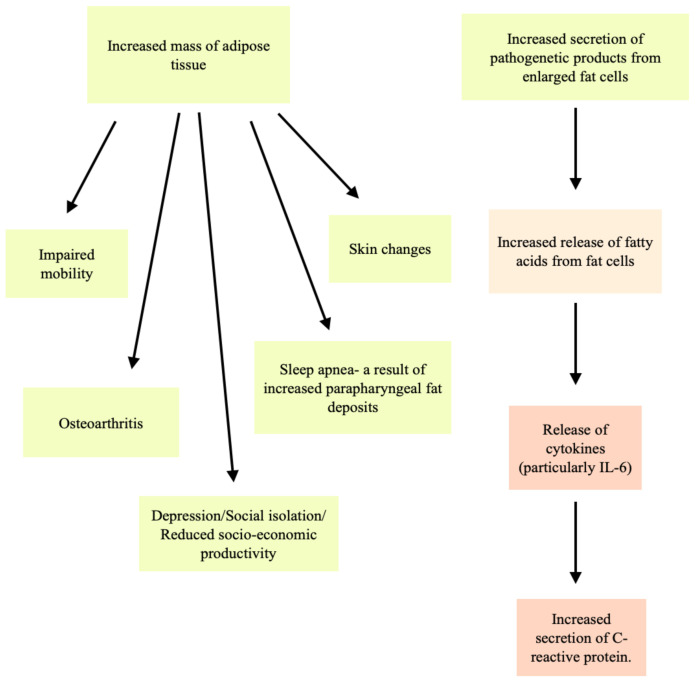
Complications resulting from the increased secretion of pathogenetic products from enlarged fat cells and the increased mass of adipose tissue.

Some of the complications associated with obesity/overweight are hypertension, atherosclerosis, diabetes, sleep apnea, osteoarthritis, and cancer [2,3,4,5,6,9]. Two main factors are considered as key points for these complications. These are the increased secretion of pathogenetic products from enlarged fat cells and the increased mass of adipose tissue (Figure 1) [4,9].

Diabetes mellitus, insulin resistance, and metabolic syndrome are considered to result from enlarged fat cells. The risk of diabetes is considered lowest in individuals with a BMI less than 22 kg/m^2^ [4]. The risk of diabetes has a linear relationship with BMI. For individuals whose BMI is equal to or greater than 35 kg/m^2^, the relative risk increases 40-fold [4].

The COVID-19 pandemic, caused by severe acute respiratory syndrome coronavirus 2 (SARS-CoV-2), broke out at a time when about 20% of humanity was overweight or obese. The number of obese or overweight individuals is increasing [7].

The COVID-19 pandemic has resulted in restriction of movement, the implementation of social distancing, and the interruption of many different sports activities. These adjustments have affected populations almost everywhere in the world by causing changes in food consumption and reduced physical activity [7]. Moreover, remote home-office environments have contributed to the increasing number of individuals with obesity [7]. A strong relationship has been found between being an individual with overweight/obesity and the risks of hospitalization and of needing treatment in intensive care units for COVID-19 [7,8,11]. Obese individuals are at significantly greater risk of acute respiratory distress syndrome, a cause of COVID-19 mortality [7].

The relationship between overweight/obesity and morbidity and mortality has been known since antiquity. The Greek physician Hippocrates said, “Sudden death is more common in those who are naturally fat than in the lean” [4]. Researchers have suggested that obesity is beginning to replace undernutrition and infectious diseases as the most significant contributor to impaired health [2].

The key factors for this epidemic of overweight and obese individuals are: increased consumption of high-energy foods, decreased physical activity, and genetic susceptibility [2,9]. There is a persistent trend of imbalance between energy intake and expenditure (a positive energy balance) in the lifestyle of many people; this results in being overweight [2,9].

Nowadays, humanity needs to find an urgent solution for the global prevalence of obesity and overweight. The current trends in the treatment of obesity/overweight include: pharmacotherapy (Orlistat, Liraglutide, Naltrexone/Bupropion), surgery, food supplements intake, diets (usually rich in proteins), and physical activity. [3,12,13,14,15]. However, these strategies often are unsuccessful in the long term.

The best strategy for reducing the number of obese/overweight individuals is the prevention of these conditions. Unfortunately, the correct formula has not yet been found.

Something relatively new in treating and preventing obesity and cardiovascular diseases is vegan and plant-based diets [15,16,17,18,19,20,21,22,23].

In the last decade, vegan and plant-based diets have been consistently associated with reduced body weight and other health benefits [16,17,18,19,20,21,22,23,24,25,26,27,28,29,30,31,32,33,34,35,36,37,38,39,40,41,42,43,44,45,46,47,48,49,50,51,52,53,54,55,56,57,58,59,60,61,62,63,64,65,66]. These diets are adopted by people mainly for health, environmental, and ethical reasons [21,22]. Researchers report that following a vegan/plant-based diet is usually associated with a healthy lifestyle that excludes smoking and includes regular physical activity. It is very likely that the health benefits from such nutritional behavior are the result of the combination of these factors, and not only the diet alone. Such a lifestyle provides many benefits and may prevent some chronic lifestyle-associated diseases, including obesity and cardiovascular diseases (CVD).

Vegan diets are dietary patterns that exclude all animals, while plant-based diets (vegetarian) do not necessarily eliminate all animal products but focus on eating mostly plants, such as fruits, vegetables, and nuts. [15,20,23,28,45]. Both diets exclude meat [15,20,28,45]. Both diets, if well balanced, are rich in fibers, vitamins, antioxidants, and carbohydrates [20]. Sufficient protein intake in the vegan diet could be provided by including foods rich in proteins such as beans, peas, chickpeas, or products that contain added plant protein [45,67].

Compared to a plant-based diet, a vegan diet provides almost no cholesterol intake [23,67]. Another difference between vegan diets, plant-based diets, and other diets is that vegan diets are able to provide the lowest calorie density [15]. Calorie density is very important for reduction in body weight. Calorie density refers to the number of kilocalories (kcal) per unit weight of food [15]. Consuming foods with lower calorie densities is more advantageous for weight loss than reducing portion sizes [15]. Foods of plant origin have lower calorie density than foods of animal origin (Table 1). In addition, food from plant sources contains no cholesterol [23,67] or only traces of cholesterol [68]. In the article “Cholesterol and Plants”, E. J. Behrman and Venkat Gopalan explain that plants may contain not only phytosterols but also cholesterol (which is considered a zoosterol). The researchers also provide the cholesterol content of some plant oils: palm oil contains up to 20 mg/kg, soybean oil up to 29 mg/kg, peanut oil up to 24 mg/kg, and sunflower seed oil up to 14 mg/kg. However, these concentrations are very low. Moreover, according to FDA rules, cholesterol quantities <2 mg per serving may be labeled as “no cholesterol” or “zero cholesterol”. E. J. Behrman and Venkat Gopalan also give a very good explanation for the cholesterol-lowering effects of phytosterols (plant sterols): phytosterols compete with cholesterol for packaging into the mixed micelles that are taken up by the polytopic transmembrane protein, Niemann-Pick C1-Like 1 (NPC1L1) [68].

Vegan diets are also associated with improved gut microbiota symbiosis, increased insulin sensitivity, reduced trimethylamine-N-oxide (TMAO), activation of peroxisome proliferator-activated receptors (PPARs), and overexpression of mitochondrial uncoupling proteins [16,24,48]. The combination of these factors might be the essence of the efficiency of this nutritional behavior. Moreover, the vegan gut profile appears to be unique in several characteristics: a reduced abundance of pathobionts, including *Enterobacteriaceae* [25,26], and a greater abundance of protective species such as *F. prausnitzii* [27].

Recent data suggest that vegan and plant-based diets are also associated with decreased all-cause mortality [28,29,31]. It was reported that these nutritional behaviors may reduce the risk of coronary heart disease (CHD) events up to 40%, the risk of cerebral vascular disease events up to 29%, and the risk of developing metabolic syndrome and diabetes by 50% [29].

It is very important for these diets to exclude processed food and food containing added sugar in order to be very well balanced; otherwise, deficiencies in proteins, ω-3 fatty acids, vitamins, and minerals can occur [21,53].

Over the past few years, the interest in vegan diets and plant-based diets has increased not only in the general population but also in the scientific community. Although current scientific data suggest that limiting the consumption of meat and dairy products may contribute to better overall health, randomized clinical trials investigating the link between plant-based diets and human well-being are limited. However, plant-based diets, and especially vegan diets, may provide many benefits not only for overweight and obese individuals but also for individuals with type 2 diabetes, CHD, or arthritis [16,17,18,19,20,21,22,23,24,25,26,27,28,29,30,31,32,33,34,35,36,37,38,39,40,41,42,43,44,45,46,47,48,49,50,51,52,53,54,55,56,57,58,59,60,61,62,63,64,65,66]. It is highly likely that in the next few years, plant-based diets may be included in some guidelines for preventing and treating cardiovascular diseases, diabetes, and obesity. Our study aimed to evaluate the association between vegan and plant-based diets and weight control.

## 2. Materials and Methods

### 2.1. Databases and Keywords

**Figure 2:**
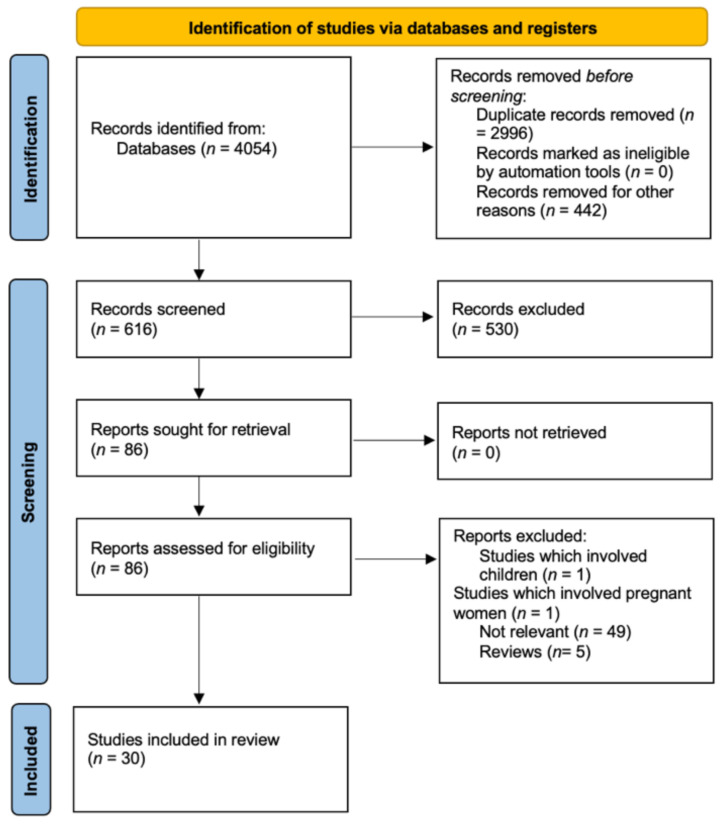
PRISMA 2020 flow diagram for new systematic reviews which included searches of databases and registers only.

Following the PRISMA guidelines for conducting systematic reviews, a search was conducted in PubMed, Web of Science, and Scopus using the following search terms: “obesity”, “globesity”, “vegan”, “plant-based diet”, “vegetarian”, “weight control”, “weight loss”, and “obesity management”. The full search strategy is reported in Figure 2 [69]. All studies were written in English. All references for the included studies were manually retrieved. All references in this manuscript were prepared with Zotero software (a project of Roy Rosenzweig Center for History and New Media).

### 2.2. Eligibility Criteria

Results were limited to original English-language articles published in full-text format in peer-reviewed journals until June 2021, assessing the direct relationship between plant-based diet and/or vegan diet and weight loss/obesity control in adults. Only clinical trials, randomized controlled trials, and prospective cohort studies were included in the results. The study selection included the screening of titles, abstracts, and full-texts and conducting a forward and backward search. All the studies selected showed a relationship between index terms “plant-based diet”, “vegetarian diet”, “vegan diet”, “obesity management”, “overweight”, and/or “weight loss”. The exclusion criteria were articles written in a language other than English, reviews, webinars, studies involving children or pregnant women, animal studies, articles with irrelevant topics, and lack of data. Inclusion criteria were human studies that investigate the relationship between obesity and vegan/plant-based diets and only adult participants.

The full search strategy is reported in Figure 2. The databases used were PubMed, Science Direct, and Web of Science up to June 2021. All studies were written in English. All the references for the included studies were manually retrieved.

## 3. Results and Discussion

In total, 8014 articles were found. After removing review articles, 4054 articles remained. After removing duplicates, 1058 articles remained. A total of 442 articles were removed for other reasons. The titles, abstracts, and full texts of 616 articles were screened for eligibility. Of the 86 articles that remained after screening, 30 references met the inclusion criteria. However, these 30 references only referred to 27 studies because two of the studies are presented in more than one article.

These studies involved a total of 2890 participants. A total of 1638 of the participants had a nutritional intervention that included a plant-based diet: vegan diet (*n* = 762)/vegetarian diet (*n* = 876).

A total of 25 (92.5%) studies presented in (Table 2) are randomized trials. A total of 17 of the studies (63%) presented in (Table 2) investigate the link between weight control and vegan diet. A total of three studies presented in (Table 2) compared vegan diet-benefits to vegetarian diet-benefits.

Although many researchers have tried to explain how these nutritional regimes impact different health conditions, many other further investigations are necessary.

### 3.1. Studies Involving Vegan Diets

A total of 17 studies investigated the relationship between a vegan diet and weight management. Most of these 17 studies were randomized trials (94%), and one study did not have a control group. A total of 14 (82.3%) studies out of the 17 vegan-trials reported body weight reduction after a vegan diet intervention [23,28,32,39,41,42,43,44,45,46,47,48,49,50,51,53,56]. Only one study reported that the BMI remained stable after a vegan intervention [52]. One study investigated the short-term benefits of a vegan intervention (changes after a vegan meal) [55], and one study compared the characteristics of vegetarians/vegans with those of omnivores [54].

According to data obtained from these trials, vegan diets, as compared to omnivorous diets, may significantly reduce body weight [23,28,32,39,41,45,46,47,50,53]. One study reported that the participants in the vegan group lost significantly more weight by the third month of the study, compared to the control group. Nevertheless, by the sixth month, there was no significant difference between groups [51]. There was no significant difference between groups when a vegetarian/vegan diet was compared to a low-fat omnivorous diet, Mediterranean diet, American Heart Association diet, or ADA diet [42,43,44,57]. It seems that all these special nutrition regimes have benefits for the reduction in body weight in overweight adults. However, plant-based diets may provide significantly reduced LDL levels compared to the Mediterranean diet [28,57]. In Table 3, we compare the differences in weight loss between vegan diet groups and other diet groups. We called the vegan groups “group I” and the other groups “group II” (low-fat omnivorous diet, “ADA” diet, Mediterranean diet, National Cholesterol Education Program diet, National Cholesterol Education Program Step II diet, and the diet recommended by the Korean Diabetes Association). The limitation of Table 3 is that the weight loss data were presented quite differently in the trials—in kilograms, as a percentage, or as reduction in BMI. The unification of the results from the different trials was not possible. However, weight loss is greater for the vegan groups. Moreover, a 2-year-long trial reported that participants in the vegan groups had successful weight control for 2 years [39].

Neal Barnard and his colleagues are among the researchers who have investigated the relationship between consumption of vegan diets and human health in many trials [23,28,30,31,39,43,44,45,46,47,48,49].

In one of these trials, they compared the effects of a vegan diet to an ADA diet for 22 weeks [23]. The researchers reported that both diets improved glycemic and lipid control in type 2 diabetic patients. However, these improvements were better in the vegan group. Moreover, the participants in the vegan group lost more body weight (6.5 kg) compared to the ADA group (3.1 kg). The waist circumference of the participants in the vegan group was reduced by –5.3 ± 4.4 cm and in the ADA group by −2.8 ± 4.7 cm. During the study, some participants needed to change their lipid-lowering medications. The researchers reported a significant reduction in LDL levels—21.2% in the vegan group compared to 10.7% in the ADA group for medication-stable patients [23].

In another study, Barnard and his team compared the vegan diet to the ADA diet for a longer period—74 weeks. The number of participants was the same as the previous study. The researchers reported no significant difference between the groups in terms of weight loss. However, weight loss was significant within each diet group: −4.4 kg in the vegan group and −3.0 kg in the other group. The lipid profile of the participants in the vegan group was reported to be better—the total cholesterol decreased by 20.4 mg/dL in the vegan group compared to 6.8 mg/dL in the other group (*p* = 0.01); LDL cholesterol decreased by 13.5 mg/dL in the vegan group compared to 3.4 mg/dL in the other group (*p* = 0.03) [43,44].

A recent study, published in 2021, compared the vegan diet to the Mediterranean diet. In general, the Mediterranean diet is considered a “healthy nutrition regime”, but many varieties of it exist. For this study, this diet included a great variety of plant-based foods and low/moderate amounts of meat, dairy products, and eggs. The main source of fat was olive oil. The study involved 62 overweight adults and was performed by Barnard and his team [28]. The study duration was 16 weeks, which is shorter than previous studies by this team [23,43,44]. Nevertheless, the study duration was sufficient for the estimation of a short-term weight loss. The researchers reported 1.5 kg weight loss in the Mediterranean diet group compared to 7.9 kg weight loss in the vegan group after 16 weeks. However, the Mediterranean diet also had some beneficial effects—the researchers reported better values of blood pressure in the Mediterranean group (the blood pressure decreased by 9.3 mm Hg systolic and 7.3 mm Hg diastolic), compared to the vegan group, for which blood pressure decreased by 3.4 mmHg systolic and 4.1 mmHg diastolic.

Yu-Mi Lee and colleagues compared the effects of a vegan diet to the diet recommended by the Korean Diabetes Association [32]. The study was a 12-week randomized clinical trial, in which participants were individuals with type 2 diabetes. The researchers reported that both diets had beneficial effects for this kind of patient—the HbA1c level decreased in both groups, by −0.5% in the intervention group (*p* < 0.01) and by −0.2% in the Korean Diabetes Association l diet group (*p* < 0.05). No significant differences in the changes in cholesterol levels were reported between the groups. However, a significant reduction in BMI and waist circumference was only reported for the vegan diet group [32].

One of the most important trials investigating the relationship between obesity/overweight lasted 2 years [39]. The duration of this study was the longest of the studies included in this manuscript. The researchers, Gabrielle M. Turner-McGrievy, Neal D. Barnard, and Anthony R. Scialli, compared the vegan diet to the NCEP diet. All participants were overweight/obese postmenopausal women. The researchers reported that the participants in the vegan group had 4.9 kg weight loss after the first year and −3.1 kg after the second year, compared to the participants in the NCEP diet group, who only had 1.8 kg weight loss after the first year and −0.8 kg after the second year.

Compared to conventional omnivorous diets, vegan diets seem to be much more effective for weight management [41,45,46,47,48,49].

Turner-McGrievy and colleagues performed a five-arm, randomized controlled trial and reported that a vegan diet is much more effective for reducing body weight than other diets, including the vegetarian diet. The weight loss in the vegan group was not only the greatest, but was also significantly different from the other four groups [41].

Kahleova and colleagues have also explored the relationship between a vegan diet and weight management by comparing vegan diet intervention to a conventional omnivorous diet. The researchers reported an average weight loss for the vegan group of 5.8 kg after 16 weeks compared to 3.8 kg for the omnivorous group [45,46,47].

Mishra and colleagues also compared the effects of an 18-week low-fat vegan diet intervention to an omnivorous diet [49]. The researchers reported an average weight loss of 2.9 kg for the vegan group and 0.06 kg for the control group. The average BMI was reduced by −1.5 kg/m^2^ for the vegan group (from 33.5 to 32 kg/m^2^). There was no reduction in BMI for the control group. The lipid profile of the vegan group was also reported to be better [49].

The role of the gut microbiota in human health is well known. It is understood that an imbalance in the microbiome may be associated with some serious pathological conditions. For example, a reduced ratio of *Bacteroidetes* to *Firmicutes* is associated with obesity [48]. In another study, Kahleova and colleagues explored the relationship between diet and human gut microbiota. The study involved 186 participants (a vegan group (*n* = 84) and a control group (*n* = 84)). The vegan group lost, on average, 5.9 kg in body weight (including 3.9 kg of fat mass), compared to 0.5 kg for the control group. The researchers not only reported changes in body weight for the vegan group, but also changes in insulin sensitivity and changes in gut microbiota composition (the relative abundance of *Faecalibacterium prausnitzii* increased) [48]. The authors suggested that changes in body weight, body composition, and insulin sensitivity in overweight adults after a low-fat vegan diet are related to changes in gut microbiota composition.

Kahleova and colleagues explored not only the effects of a long-term nutritional intervention, but also the direct effects of a vegan meal compared to the direct effects of a meat meal [55]. The study had a randomized crossover design. The researchers analyzed brain activity, gastrointestinal hormones, and satiety after the intake of vegan/meat meals. The participants were individuals with type 2 diabetes (*n* = 20), overweight/obese adults (*n* = 20), and healthy adults (*n* = 20). Before the intervention, the participants had a fasting period of 10–12 h overnight. The participants with type 2 diabetes had to skip their diabetes medication in the evening and morning before the intervention. Measurements were taken at baseline (time 0) and then 180 min after the meal intake. The washout period between both test meals was 1 week. The researchers reported very interesting findings: the insulin secretion was higher after the vegan meal, compared to the meat meal, for the participants with diabetes. After the vegan meal, insulin sensitivity was the highest in overweight/obese adults compared with participants with diabetes and healthy adults. Compared to the meat meal, insulin sensitivity increased in all groups after the vegan meal by more than 40% [55].

Compared to other diets, including vegetarian diets, the vegan diet seems to be much more beneficial in terms of weight control [23,28,32,39,41,43,44,45,46,47,49,51,53,56], reducing total cholesterol and LDL levels [42,43,44], and decreasing levels of fasting blood glucose, fasting insulin and insulin resistance [42,43,44]. This nutritional behavior could be very useful in the prevention and treatment of certain diseases and could be included in the lifestyle of individuals with type 2 diabetes, cardiovascular diseases, or overweight.

In our view, the key points of the mechanism by which the vegan diet has a beneficial effect on conditions such as obesity and overweight are:

1. Lower calorie density compared to other diets—individuals can eat bigger portions because plants have a lower calorie density than foods of animal origin (Table 1).
2. Antioxidants—vegan and plant-based diets, if well balanced, contain plentiful fruits and vegetables, which are rich in antioxidants. The consumption of raw fruits, vegetables, roots, nuts, and germinated seeds provides an intake of carotenoids, vitamin C, vitamin E, and other compounds that have an antioxidant effect.
3. Lipid-lowering effects—the absence [23,67] or limited intake of dietary cholesterol [68]. Moreover, some plants that are rich in sterols and stanols may lower serum low-density lipoprotein cholesterol concentrations and improve endothelial dysfunction [37,38]. Foods rich in sterols/stanols are nuts, flaxseed, fresh cauliflower (200 mg/100 g), avocado (75 mg/100 g), raspberry, lingonberry, grapes, apple, blueberry, and others [38]. Potatoes are an example of a poor source of sterols/stanols [38].

Most obese/overweight adults are diagnosed with atherosclerosis, which is considered one of the most important risk factors for ischemic heart disease and ischemic stroke [70,71,72,73]. For that reason, plant-based diets may have an important role in the future management not only of obesity/overweight but also in the management of CVD. Many studies provided evidence that vegan diets have a strong relationship with reducing the risk of CVD by reducing the total cholesterol levels and LDL levels [23,42,43,44,49,50].

However, the reduction in LDL levels is not the only key factor that reduces the risk of CVD. There is another mechanism by which plant-based/vegan diets might reduce the risk of major adverse cardiovascular events. This mechanism involves a reduction in the concentration of C-reactive protein, which is considered an important marker of inflammation [74]. Not only is the relationship between chronic inflammation and the progression of atherosclerosis well known [42,75,76,77,78,79,80,81], but targeted anti-inflammatory therapy and reductions in C-reactive protein have also previously been shown to reduce adverse cardiovascular events in patients with already established coronary artery disease [42,81].

Binita Shah and colleagues studied the anti-inflammatory effects of a vegan diet compared to the American Heart Association diet [42]. The study included 100 participants with established coronary artery disease, who were assigned to follow either a vegan diet (*n* = 50) or the American Heart Association diet (*n* = 50). The study duration was 8 weeks. The researchers reported not only better anthropometric data for the participants in the vegan group (the mean BMI was reduced from 30.5 at baseline to 29; the mean waist circumference was reduced from 107 cm at baseline to 102 cm at week 8) but also better glycemic markers, better lipid profile, and reduction in the concentrations of C-reactive protein. The total cholesterol decreased from 136 mg/dL (mean value at baseline) to 127 mg/dL (mean value at week 8) in the vegan group compared to a reduction from 146 mg/dL (mean value at baseline) to 142 mg/dL (mean value at week 8) in the American Heart Association group.

Overweight people suffer from a great variety of CVD, type 2 diabetes, joint pain (because of increased body mass), and other health problems. During our research for the link between plant-based diets and the prevention of obesity/overweight, we discovered studies that report that vegan diets are beneficial for recovery in patients with osteoarthritis. Kjeldsen-Kragh and colleagues performed a randomized, single-blind controlled trial of fasting and a one-year vegetarian diet in rheumatoid arthritis [33]. The study included 53 participants, randomized into two groups. A total of 27 adults were put on an individually adjusted, gluten-free, vegan diet for 3.5 months. After 3.5 months, their diet was changed to a vegetarian diet for the remaining period of the study. The control group included 26 patients who had their ordinary diet throughout the whole study period. After 4 weeks, the vegan diet group showed a significant improvement in the number of swollen joints, pain score, duration of morning stiffness, C-reactive protein, etc. These benefits were reported to have a long-term effect. The authors reported that the patients in the diet group lost more weight than the control group. The authors reported that the evaluation of the whole course showed significant advantages for the diet group in all measured indices.

The data from this research indicate that plant-based diets may benefit overweight individuals with joint problems. This study was not included in Table 2, which represents the link between plant-based diets and weight loss, because the study had another focus, and it did not include specific information about weight loss. However, the study represents important data that may be used in different therapeutic strategies.

Other studies also provide evidence that plant-based diets may improve the signs and symptoms of rheumatoid arthritis [34,35,36].

**Figure 3:**
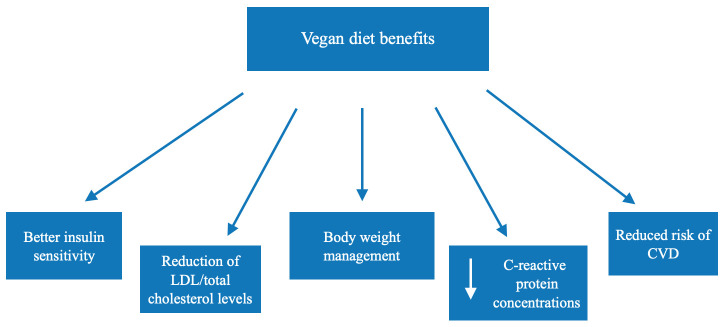
Beneficial effects associated with vegan diets.

Weight loss/obesity management should not only include strategies for reducing body mass but also for reaching a better overall health status, including reduced cholesterol levels, reduced triglyceride levels, and better glycemic control. That is why a vegan diet could be especially useful in the prevention/treatment of not only obesity/overweight but also CVD (Figure 3).

However, a vegan diet should be well planned and balanced and should exclude processed foods. The intake of food supplements could support this type of diet to avoid the deficiency of some vitamins and minerals. It is important to include B12 supplementation because its deficiency is commonly associated with such nutritional regimes [53,56,82,83]. Vitamin D supplementation is also beneficial for vegan diets because it is mainly present in foods of animal origin.

### 3.2. Studies Involving Vegetarian Diets

We have included 11 studies that investigate the relationship between vegetarian diets and obesity/overweight management [40,41,57,58,59,60,61,62,63,64,66]. All studies associated the vegetarian diet with better overall health including reductions in LDL cholesterol, total cholesterol, and fasting insulin levels. However, the results concerning the reduction in body weight vary from no significant reduction [64] up to 10 kg weight loss.

D. Ornish and colleagues performed one of the earliest clinical trials investigating if lifestyle changes (including following a low-fat vegetarian diet, stopping smoking, and moderate physical activity) can affect coronary atherosclerosis [59]. The study was a prospective, randomized, controlled trial, and included an experimental group (*n* = 28) and a usual-care control group (no interventions) (*n* = 20). The results were in favor of the experimental group. The participants in the experimental group had a better lipid profile (LDL—3.93 mmol/L at baseline and 2.46 mmol/L at the first year) compared to the control group profile (LDL—4.32 mmol/L at baseline and 4.07 mmol/L at the first year). The average blood pressure also decreased in the experimental group (134/83 at baseline and 127/79 at first year). The participants in the experimental group reported a reduction in the frequency of chest pain by 91%, reduction in chest pain duration by 42%, and reduction in the severity of angina by 28%. However, these values worsened for the control group—chest pain frequency did not reduce but more than doubled, and the angina duration increased from 3.47 min to 6.97 min. The authors reported that the diameter of stenosis regressed significantly in the experimental group and progressed in the control group. The mean weight loss of the experimental group was 10 kg. The mean weight of the control group was 80.4 kg at the beginning of the study and 81.8 kg at the end of the study. This study demonstrated the strength of the relationship between plant-based diets, healthy lifestyles, and CVD management.

The researchers made an important statement—lifestyle changes may cause not only significant weight loss but also a regression of even severe coronary atherosclerosis without the use of lipid-lowering drugs after only one year [59]. Therefore, a plant-based diet should be widely recommended for patients with CVD.

Another very important study reported on the relationship between the plant-based diets and s better lipid profile [66]. Bunner and colleagues explored the effects of a 20-week plant-based intervention on individuals with type 2 diabetes. The intervention group included 17 participants, and the control group also included 17 participants. The total cholesterol declined by 12.1 mg dL^−1^ in the intervention group and by 2.2 mg dL^−1^ in the control group. LDL cholesterol declined by 7.8 mg dL^−1^ in the intervention group and increased by 0.4 mg dL^−1^ in the control group. The average body weight decreased by 7 kg for the plant-based group, compared to 0.6 kg for the control group [66].

However, the vegan diet seems to be much more beneficial in terms of cholesterol reduction. Vegetarian diets were not always able to provide a significant reduction in cholesterol levels [58].

Some other beneficial changes in health status, which were associated with vegan diets, were also associated with vegetarian diets including but not limited to: healthier microbiota composition [63], a significant decrease in C-reactive protein [60,61], a better glycemic profile [60,62], and better values of blood pressure [40,60].

## 4. Conclusions

The relationship between overweight/obesity, CVD, and diabetes is well known and extensively discussed. Current scientific data suggest that limiting the consumption of meat and dairy products may contribute to better overall health. However, randomized clinical trials investigating the link between plant-based diets and human well-being are limited. Plant-based diets, and especially vegan diets, may provide many benefits not only for overweight and obese individuals but also for individuals with type 2 diabetes, CVD, and arthritis.

It is very important for the nutritional management of overweight/obesity to provide not only a reduction in body mass but also a better overall health status, including reduced cholesterol levels, reduced triglyceride levels, and better glycemic control.

For that reason, a plant-based diet could become a novel successful instrument to fight the pandemic of “globesity”. However, long-term clinical studies should be carried out to establish the relationships between vegan diets, the prevention and treatment of obesity, and certain chronic non-communicable degenerative diseases. It is highly likely that, in the near future, these diets will be included in the therapy guidelines for of type 2 diabetes, CVD, obesity, and overweight.

## Figures and Tables

**Table 1:** Average kilocalories, cholesterol, and protein content in some foods used in different types of diets.

Food	Average Kilocalories (kcal) Per 100 g	Average Cholesterol Content mg/100 g	Average Protein Content g/100 g	Omnivorous Diet	Plant Based Diet	Vegan Diet
Ground beef	260	87	25.54	+	−	−
Beef (roast)	219	82	27.45	+	−	−
Beef sausage	328	61	13.3	+	−	−
Chicken feet	215	84	19.4	+	−	−
Chicken (back)	298	87	25.73	+	−	−
Pork hash	185	56	12.96	+	−	−
Pork sausage	325	86	18.53	+	−	−
Fish	188	90	21.74	+	−	−
Eggs* (hard-boiled)	155	373	12.6	+	+	−
Hard cheese	157	69	13.83	+	+	−
Cheese dip	160	8	3.24	+	+	−
Feta cheese	265	89	14.21	+	+	−
Roquefort cheese	353	75	21.4	+	+	−
Brie cheese	334	100	20.75	+	+	−
Cow milk (whole)	67	15	3.3	+	+	−
Cow butter, light	499	106	3.3	+	+	−
Peanut butter	597	0	22.5	+	+	+
Almond butter	614	0	20.94	+	+	+
Corn oil	900	0	0	+	+	+
Coconut oil	883	0	0	+	+	+
Olive oil	884	0	0	+	+	+
Sesame oil	884	0	0	+	+	+
Sunflower oils	884	0	0	+	+	+
Soy milk (unsweetened)	38	0	3.53	±	±	+
Oat milk	50	0	1.25	±	±	+
Beyond meet R- beyond burger	230	0	17.7	±	+	+
Beyond meet R- beyond chicken grilled strips	141	0	23.53	±	+	+
Beyond meet R- plant-based brat	280	0	18.67	±	+	+
Mushrooms (raw)	22	0	3.09	+	+	+
Potatoes, baked	93	0	1.95	+	+	+
Chickpeas (canned)	128	0	8	+	+	+
Green peas (frozen)	79	0	5.62	+	+	+
Arugula (raw)	25	0	2.58	+	+	+
Tomatoes (raw)	22	0	0.88	+	+	+
Red peppers (raw)	17	0	0.68	+	+	+
Spinach (raw)	23	0	2.86	+	+	+
Cauliflower (raw)	25	0	1.92	+	+	+
Cucumbers (raw)	10	0	0.59	+	+	+
Avocado (raw)	160	0	2	+	+	+
Melon (honeydew, raw)	36	0	0.54	+	+	+
Watermelon (raw)	30	0	0.61	+	+	+
Blueberries (raw)	57	0	0.74	+	+	+
Strawberries (raw)	32	0	0.67	+	+	+
Rose-apples (raw)	25	0	0.6	+	+	+

* Egg: the data are presented per 100 g (usually one small egg is 50 g. The symbol “−” in the table means “absent in the specific diet”. The symbol “+” in the table means “present in the specific diet”. Data obtained from the USDA National Nutrient Database for Standard Reference [67].

**Table 2:** Studies involving the relationship: plant-based diets–obesity/overweight.

Study Design	No of P. *	Study Duration	Diets That Were Studied	Main Results	Ref.
Participants with type 2 diabetes were randomly assigned to a low-fat vegan diet or a diet following the American Diabetes Association (ADA) guidelines.	99	22 weeks	Vegan (*n* = 49)/American Diabetes Association (ADA) guidelines (*n* = 50)	A total of 43% of the vegan group and 26% of the ADA group participants reduced usage of diabetes medications. Both diets improved glycemic and lipid control. However, these improvements were reported to be better with a vegan diet.	[23]
A randomized crossover trial, which included overweight adults assigned to 2 groups.	62	16 weeks	Vegan diet (*n* = 30)/Mediterranean diet (*n* = 32)	Significant reduction in body weight in the vegan group.	[28]
A randomized trial, which included participants diagnosed with type 2 diabetes randomly assigned into 2 groups.	92	12 weeks	Vegan diet (*n* = 46)/Korean Diabetes Association Diet (*n* = 46)	A significant reduction in HbA1C levels was reported for both groups. However, glycemic control was found to be better with the vegan diet than with the conventional diet.	[32]
A randomized trial which included overweight, postmenopausal women randomly assigned into 2 groups.	62	2 years	A low-fat, vegan diet (*n* = 28)/National Cholesterol Education Program diet (*n* = 34)	Individuals in the vegan group lost significantly more weight than those in the National Cholesterol Education Program group at 1 year (−4.9 (−0.5, −8.0) kg vs. −1.8 (0.8, −4.3); *p* < 0.05) and at 2 years (−3.1 (0.0, −6.0) kg vs. −0.8 (3.1, −4.2) kg; *p* < 0.05).	[39]
The study analyzed the interaction between BMI and vegetarian status. This was tested using a multivariable regression analysis adjusting for age, education, smoking, alcohol drinking, and physical activity.	170	No specific period	Vegetarian (*n* = 170)/omnivore diet (*n* = 120)	Compared with omnivores, vegetarians had significantly lower mean levels of BMI, blood pressure, total cholesterol, LDL cholesterol, and triglycerides. The researchers suggested a lower predicted probability of coronary heart disease for vegetarians.	[40]
A five-arm, randomized controlled trial, in which participants were overweight adults (BMI: 25.0–49.9), 18–65 years old. All tested diets were low-fat, which included limited amounts of nuts, butters, avocado, seeds, and olives.	63	6 months	Vegan (*n* = 12)/vegetarian (*n* = 13)/pesco-vegetarian (*n* = 13)/semi-vegetarian (*n* = 13)/omnivorous (*n* = 12)	At the 6th month, the weight loss in the vegan group (−7.5% ± 4.5%) was significantly different from the other groups.	[41]
Open-label, blinded end-point randomized trial that included participants with coronary artery disease. The participants were randomized into 2 groups.	100	8 weeks	Vegan (*n* = 50)/American Heart Association diet (*n* = 50)	A vegan diet resulted in a significantly (32%) lower high-sensitivity C-reactive protein compared with the American Heart Association diet. The degree of reduction in body mass did not significantly differ between the 2 diet groups.	[42]
A controlled trial in which participants were individuals with type 2 diabetes. Participants were randomly assigned into 2 groups.	99	74 weeks	Vegan (*n* = 50)/American Diabetes Association guidelines (ADA) (*n* = 50)	Both groups reported reduced hunger and reduced disinhibition. The mean weight loss was reported to be 22% for the vegan group in week 74 and 20% for the ADA group.	[43,44]
A randomized clinical trial. Dual-energy X-ray absorptiometry was used to measure body composition. Insulin resistance was assessed with the Homeostasis Model Assessment index.	75	16 weeks	Vegan (*n* = 38)/omnivorous diet (*n* = 37)	Weight decreased significantly in the vegan group. The mean weight loss for the vegan group was 5.8 kg compared to 3.8 kg for the omnivorous group.	[45,46,47]
A single-center, randomized, open, parallel design. All participants had a BMI between 28 and 40 kg/m^2^. Gut microbiota composition was assessed using uBiome Explorer™ kits; body composition and insulin sensitivity were also measured.	168	16 weeks	Vegan diet (*n* = 84)/omnivorous diet (*n* = 84)	The data suggested that the low-fat vegan diet led to an increased relative abundance of *Faecalibacterium prausnitzii* and a smaller decrease, compared to the control group, in the relative abundance of *Bacteroides fragilis.* Changes in the relative abundance of *Bacteroides* *fragilis* were found to correlate positively with changes in insulin sensitivity. Body weight was significantly reduced in the vegan group (treatment effect: –5.9 kg).	[48]
A multicenter randomized control trial.	291	18 weeks	Vegan diet (*n* = 142)/omnivorous diet (*n* = 149)	Authors reported improved body weight, plasma lipids, and glycemic status for the vegan group.	[49]
A parallel design study in which participants were overweight hyperlipidaemic men and postmenopausal women.	39	6 months	Vegan diet (*n* = 20)/vegetarian diet (*n* = 19)	The relative LDL cholesterol and triglyceride reductions were found to be greater in the vegan group.	[50]
A randomized study that only included overweight/obese women with polycystic ovary syndrome.	18	6 months	Vegan diet (*n* = 9)/low-cal. diet (*n* = 9)	The participants in the vegan group lost significantly more weight by the third month. However, there was no difference between groups at 6 months.	[51]
A randomized, controlled trial. The participants were randomized into 2 groups. All participants had a pre-treatment phase consisting of a 1-week, controlled, mixed diet.	53	4 weeks	Vegan diet (*n* = 26)/meat-rich diet (*n* = 27)	In the vegan group, the total leukocyte, neutrophil, monocyte, and platelet counts decreased, and after 4 weeks, they were significantly lower than the other group.	[52]
A randomized, controlled trial that included overweight postmenopausal women.	59	14 weeks	Low-fat vegan diet (*n* = 29)/National Cholesterol Education Program Step II diet (*n* = 30)	The low-fat vegan diet was associated with greater decreases in fat, saturated fat, protein, and cholesterol intake than the other diet. In both groups, there was a significant reduction in BMI. There was a significant difference between the groups.	[53]
The study compared some parameters of vegan/vegetarians to omnivores. Laboratory tests were performed for fasting blood glucose and fasting insulin concentrations.* The vegan/vegetarian participants were people who had followed this diet for at least 1 year.	558	No specific duration	Vegetarian diet (*n* = 206) and vegan diet (*n* = 73)/omnivore diet (*n* = 279)	Authors reported that a vegan diet was associated with lower fasting blood glucose, fasting insulin, and insulin resistance.	[54]
A randomized crossover study. The participants were men diagnosed with type 2 diabetes, overweight/obese men, and healthy men as the control. Participants with type 2 diabetes were instructed to skip their diabetes medication in the evening and morning before the assessments. The meals consisted of either a conventional meat cheeseburger or a plant-based tofu burger.	60	No specific duration	Vegan meal/meat-containing meal	Authors reported higher postprandial GLP-1 secretion after the vegan meal in men with type 2 diabetes, greater satiety, and changes in thalamus perfusion. Authors suggested a potential use of plant-based meals in addressing the key pathophysiologic mechanisms of food intake regulation.	[55]
The researchers studied the effect of a vegan diet on nutrient intake, body weight, and mood. No control group.	16	30 days	Vegan diet	The authors reported average weight loss of 1.7 kg.	[56]
A randomized dietary intervention trial. The participants were divided into 2 groups. The dietary profile of both groups included the average intake of 2071.3± 548.4 kcal/day.	118	3 months	Vegetarian diet (*n* = 60)/Mediterranean diet (*n* = 58)	LDL levels were significantly reduced in the vegetarian group. Both diets were effective in reducing body weight, BMI, and fat mass. However, there were no significant differences between the groups for these parameters.	[57]
A randomized clinical trial. Both diets were calorie-restricted and low-fat.	176	1 year + 6 months maintenance phase	A vegetarian diet (*n* = 96)/standard diet (*n* = 80)	All participants had a reduction in total energy and fat intake and an increase in energy expenditure. This was reflected in reduced body weight. An insignificant decrease in LDL cholesterol levels for the vegetarian group was reported (*p* = 0.06).	[58]
A randomized, controlled trial that aimed to determine whether comprehensive lifestyle changes can affect coronary atherosclerosis after 1 year. Participants were divided into two groups. The first group was called “the experimental group” and had a low-fat vegetarian diet and some other lifestyle changes, including stopping smoking, stress management training, and moderate exercise. The control group had no lifestyle changes.	48	1 year	A low-fat vegetarian diet (*n* = 28)/omnivorous diet (20)	In the experimental group, the total cholesterol levels were reduced from 5.88 mmol/L to 4.45 mmol/L; the LDL levels were reduced from 3.92 mmol/L to 2.46 mmol/L. In the control group, the total cholesterol levels were reduced from 6.34 mmol/L to 6.00 mmol/L, while the LDL levels were reduced from 4.32 mmol/L to 4.07 mmol/L. The mean weight of the experimental group was 91.1 kg at the beginning of the study and was reduced to 81 kg. The mean weight of the control group was 80.4 kg at the beginning of the study and 81.8 kg at the end of the study.	[59]
A prospective cohort study. The participants were people with coronary heart disease (CHD) or individuals at high risk with >3 CHD risk factors and/or diabetes. The intervention included a plant-based diet, moderate physical activity, and stress management.	131	3 months	Plant-based diet/no control group	All participants had improved health status after 3 months of the study. Researchers reported significant reduction in: BMI, systolic and diastolic blood pressure, waist/hip ratio, C-reactive protein, insulin, LDL, and total cholesterol. The mean BMI was reduced from 33.6 to 31.8 kg/m^2^ (*p*-value < 0.001). The quality of life and cognitive functioning were also improved.	[60]
Multisite cardiac lifestyle intervention program. The participants were individuals with coronary artery disease (CAD) and/or risk factors for CAD. The control group had the usual standard of care, while the experimental group had a lifestyle intervention: a plant-based diet, moderate physical activity, stress management, and smoking cessation.	47	12 weeks	Plant-based diet (*n* = 27)/control group (*n* = 20)	The mean body weight of the experimental group was 96.2 ± 3.8 kg at the baseline and 90.7 ± 3.6 kg at the end of the study. The mean body weight of the control group was 90.7 ±3.5 kg at the baseline and 91.2 ± 3.4 kg at the third month of the study. A significant decrease in C-reactive protein and interleukin-6 was reported in the experimental group.	[61]
A randomized, controlled trial. Participants were individuals with multiple sclerosis. The mean BMI was 28.4 ± 6.76 kg/m^2^ for the control group and 29.3 ± 7.42 for the diet group.	61	1 year	A very-low-fat, plant-based diet (*n* = 32)/control group (*n* = 29)	Authors reported a significant reduction in BMI in the diet group, which was an average of 0.18 kg/m^2^ per month or 0.5 kg per month. The plant-based diet intervention also benefited the self-reported outcome of fatigue and reductions in LDL cholesterol, total cholesterol, and fasting insulin levels.	[62]
A randomized pilot study that evaluated the effect of 45-days of 3 types of isocaloric very-low-calorie ketogenic diets on the microbiota in patients with obesity and insulin resistance. The mean BMI of the participants was 35.9 ± 4.1 kg/m^2^. The participants were randomly assigned to 3 groups. The diets included 780 kcal/day.	48	45 days	A diet with whey protein (*n* = 16)/a diet with vegetable protein (*n* = 16)/a diet with animal protein (*n* = 16)	Authors reported a significant reduction in initial body weight both in the whey protein group and in the vegetable protein group. Although a decreasing trend in total fat and trunk fat mass was observed in the three groups, a significant difference was observed only in the whey protein group and vegetable protein group. It was reported that following a plant-based very-low-calorie ketogenic diet is associated with healthier microbiota composition.	[63]
A randomized controlled trial. The aim of the study was to compare the effect of a standard calorie- and fat-restricted diet vs. a lacto-ovo-vegetarian diet on total and high-molecular-weight and on total adiponectin levels. The participants were overweight/obese adults.	143	6 months	Standard diet (*n*= 79)/vegetarian diet (*n*= 64).	A significant weight loss was reported in both groups (no significant differences between the groups).	[64]
A randomized pilot study in which participants were individuals with type 2 diabetes and painful diabetic neuropathy. The participants were randomly assigned to 2 groups. The participants’ mean age was 57 years. The mean BMI was 36 kg/m^2^.	34	20 weeks	A low-fat, plant-based diet (*n* = 17)/omnivorous diet (*n* = 17)	The body weight was reduced by a mean of 7.0 kg over 20 weeks in the intervention group. In the control group, the mean weight loss was 0.6 kg.	[66]

* No of P.—number of participants.

**Table 3:** Studies comparing vegan diets to other diets, which are specially developed for management of some medical conditions or for weight control.

Number of the Participants in Group I	Number of the Participants in Group II	Study Duration	Mean Weight Loss in Group I/Reduction in BMI	Mean Weight Loss in Group II/Reduction in BMI	Ref.
49	50 (ADA)	22 weeks	Body weight decreased by 6.5 kg.	Body weight decreased by 3.1 kg.	[23]
49	50 (ADA)	74 weeks	The mean weight loss was 22%.	The mean weight loss was 20%.	[43,44]
30	32 (Mediterranean diet)	16 weeks	The mean weight loss was 7.9 kg.	The mean weight loss was 1.5 kg.	[28]
46	47 (a diet by Korean Diabetes Association)	12 weeks	Reduction in BMI with −0.5 ± 0.9.	Reduction in BMI with −0.1 ± 0.6.	[32]
28	34 (NCEP diet)	2 years	−4.9 kg at the first year and −3.1 kg at the second year.	−1.8 kg at the first year and −0.8 kg at the second year.	[39]
50	50 (American-Heart- Association-recommended diet)	8 weeks	BMI index was reduced from 30.5 to 29.0 kg/m^2^.	BMI index was reduced from 30.9 to 29.5 kg/m^2^.	[42]
9	9 (a low-cal. omnivorous diet)	3 mounts	1.8% weight loss in the first 3 mounts.	0.0% weight loss in the first 3 mounts.	[51]
29	30 (National Cholesterol Education Program Step II diet)	14 weeks	BMI index reduced from 33.6 ± 5.2 to 31.5 ± 5.2 kg/m^2^.	BMI index reduced from 32.6 ± 3.3 to 31.2 ± 3.5 kg/m^2^.	[53]

## Data Availability

Not applicable.
